# Sodium‐glucose cotransporter 2 inhibitors versus dipeptidyl peptidase 4 inhibitors on new‐onset overall cancer in Type 2 diabetes mellitus: A population‐based study

**DOI:** 10.1002/cam4.5927

**Published:** 2023-05-06

**Authors:** Cheuk To Chung, Ishan Lakhani, Oscar Hou In Chou, Teddy Tai Loy Lee, Edward Christopher Dee, Kenrick Ng, Wing Tak Wong, Tong Liu, Sharen Lee, Qingpeng Zhang, Bernard Man Yung Cheung, Gary Tse, Jiandong Zhou

**Affiliations:** ^1^ Diabetes Research Unit Cardiovascular Analytics Group, China‐UK Collaboration Hong Kong China; ^2^ Division of Clinical Pharmacology and Therapeutics, Department of Medicine, LKS Faculty of Medicine The University of Hong Kong Hong Kong China; ^3^ Department of Radiation Oncology Memorial Sloan Kettering Cancer Center New York New York USA; ^4^ Department of Medical Oncology University College London Hospitals NHS Foundation Trust London UK; ^5^ School of Life Sciences Chinese University of Hong Kong Hong Kong China; ^6^ Tianjin Key Laboratory of Ionic‐Molecular Function of Cardiovascular Disease, Department of Cardiology, Tianjin Institute of Cardiology Second Hospital of Tianjin Medical University Tianjin China; ^7^ School of Data Science City University of Hong Kong Hong Kong China; ^8^ Kent and Medway Medical School University of Kent and Canterbury Christ Church University Canterbury UK; ^9^ School of Nursing and Health Studies Hong Kong Metropolitan University Hong Kong China; ^10^ Nuffield Department of Medicine University of Oxford Oxford UK

## Abstract

**Background:**

Cancer is currently the second leading cause of death globally. There is much uncertainty regarding the comparative risks of new‐onset overall cancer and pre‐specified cancer for Type 2 diabetes mellitus (T2DM) patients on sodium‐glucose cotransporter 2 inhibitors (SGLT2I) versus DPP4I.

**Methods:**

This population‐based cohort study patients included patients who were diagnosed with T2DM and administered either SGLT2 or DPP4 inhibitors between 1 January 2015 and 31 December 2020 in public hospitals of Hong Kong.

**Results:**

This study included 60,112 T2DM patients (mean baseline age: 62.1 ± 12.4 years, male: 56.36%), of which 18,167 patients were SGLT2I users and 41,945 patients were dipeptidyl peptidase 4 inhibitor (DPP4I) users. Multivariable Cox regression found that SGLT2I use was associated with lower risks of all‐cause mortality (HR: 0.92; 95% CI: 0.84–0.99; *p*= 0.04), cancer‐related mortality (HR: 0.58; 95% CI: 0.42–0.80; *p* ≤ 0.001) and new diagnoses of any cancer (HR: 0.70; 95% CI: 0.59–0.84; *p* ≤ 0.001). SGLT2I use was associated with a lower risk of new‐onset breast cancer (HR: 0.51; 95% CI: 0.32–0.80; *p* ≤ 0.001), but not of other malignancies. Subgroup analysis on the type of SGLT2I, dapagliflozin (HR: 0.78; 95% CI: 0.64–0.95; *p* = 0.01) and ertugliflozin (HR: 0.65; 95% CI: 0.43–0.98; *p* = 0.04) use was associated with lower risks of new cancer diagnosis. Dapagliflozin use was also linked to lower risks of breast cancer (HR: 0.48; 95% CI: 0.27–0.83; *p* = 0.001).

**Conclusion:**

Sodium‐glucose cotransporter 2 inhibitor use was associated with lower risks of all‐cause mortality, cancer‐related mortality and new‐onset overall cancer compared to DPP4I use after propensity score matching and multivariable adjustment.

## Abstract 2

SGLT2I was associated with lower risk of all‐cause mortality, cancer‐related mortality and new‐onset overall cancer compared to DPP4I after propensity score matching and multivariable adjustment.

## 1 INTRODUCTION

The burden of cancer incidence has drastically increased over the years and is currently the second leading cause of death globally. In 2020, the Global Cancer Observatory estimated a total of 19.3 million new cancer cases and 10 million cancer deaths.^1^ Despite efforts to advance preventive interventions, the asymptomatic nature of the disease during its early stages poses a challenge for cancer diagnosis.^2^, ^3^ Although the aetiology of some cancer types still requires further exploration, currently established risk factors include but are not limited to Type 2 diabetes mellitus (T2DM), hypertension and smoking.^4^ Numerous epidemiological studies have found supporting evidence for the association between T2DM and many different types of cancer, such as liver cancer, breast cancer and colorectal cancer.^5^, ^6^ As such, this has generated growing interest into anti‐diabetic medications as a potential adjuvant in the clinical management of cancer.

Metformin, in multiple pre‐clinical studies, has been described to be useful in the treatment of various types of malignancies.^7^, ^8^, ^9^ However, current evidence presents conflicting results regarding the use of novel anti‐diabetic agents such as sodium‐glucose cotransporter 2 inhibitors (SGLT2I) and dipeptidyl peptidase 4 inhibitors (DPP4I). A systematic review and meta‐analysis revealed canagliflozin had protective effects against gastrointestinal cancers, while empagliflozin was found to have increased risks of bladder cancer.^10^ Similarly, previous studies have reported increased risks of liver, kidney and bladder cancer and melanoma in T2DM patients using DPP4I.^11^ In stark contrast, there is also evidence to suggest the absence of any association between these medications and malignancy, even when stratified by different subtypes of DPP4I.^12^ Regarding SGLT2I, a retrospective study from Taiwan found SGLT2I usage was associated with lower risks of cancer‐related mortality relative to DPP4I.^13^ Likewise, another investigation comparing the risk of urinary tract and haematological malignancies amongst SGLT2I and DPP4I users demonstrated superiority of the former.^14^

Despite the aforementioned findings, there is still much uncertainty regarding the comparative associations between SGLT2I and DPP4I with different types of new‐onset overall cancer.^15^, ^16^ Given the prevalence with which these medications are used, the present study aims to assess the effects of SGLT2I versus DPP4I on the risk of new‐onset overall cancer and pre‐specified cancers in T2DM patients from Hong Kong.

## 2 METHOD

### 2.1 Study population

This population‐based, retrospective study has assessed integrated medical records of patients through the Clinical Data Analysis and Reporting System (CDARS), including disease diagnosis, laboratory results, past comorbidities, medication prescription details and clinical characteristics. The system has also been used by our team in previous epidemiological research in Hong Kong.^17^, ^18^, ^19^ Patients who were diagnosed with T2DM and were administered either SGLT2 or DPP4 inhibitors, between 1 January 2015 and 31 December 2020, in centres under the Hong Kong Hospital Authority were included in the study cohort. The exclusion criteria for the cohort were as follows: (1) patients who died within 30 days after initial drug exposure; (2) patients under 18 years old; (3) patients with prior all‐cause malignancies; (4) patients with new‐onset all‐cause malignancies development less than 1 year after drug exposure; and (5) patients with both DPP4I and SGLT2I prescription. The study has received Ethics Approval from The Joint Chinese University of Hong Kong‐New Territories East Cluster Clinical Research Ethics Committee (Application reference: 2018.643, 2018.309).

### 2.2 Clinical and biochemical data collection

Biochemical and clinical data were extracted for this cohort. Patients' demographic information includes sex, baseline age and date of initial drug use. Past comorbidities include diabetes mellitus disease duration, hyperlipidaemia, obesity, hypertension, alcoholism, liver diseases, autoimmune diseases, HIV, carcinogen pathogens, previous irradiation, chronic obstructive pulmonary disease, gastrointestinal diseases, cardiovascular diseases, ischemic stroke, diabetic eye diseases and renal diseases. Moreover, Charlson's standard comorbidity index was also calculated. Renal function was calculated using the CKD‐EPI equation.^20^

Moreover, anti‐diabetic and non‐SGLT2I/DPP4 medications and baseline laboratory data results were also extracted. Data on the following medications were extracted: sulphonylurea, insulin, metformin, thiazolidinedione, acarbose, glucagon‐like peptide‐1 receptor agonists, statins and fibrates, Angiotensin‐converting enzyme inhibitors, Angiotensin receptor blockers, anti‐depressant drugs, antihypertensive drugs, anti‐hepatitis drugs, anticoagulants, diuretics, nitrates, beta‐blockers, calcium channel blockers and non‐steroidal anti‐inflammatory drugs. The extracted laboratory data include lipid profiles, complete blood count, renal function test, biochemical test and glycaemic profiles.

### 2.3 Outcome and statistical analysis

The primary outcome of this study was new‐onset all‐cause cancer incidence, all‐cause cancer‐related mortality and all‐cause mortality. Mortality data were extracted from the Hong Kong Death Registry, an official government registry linked with CDARS that registers death records of all Hong Kong citizens. Study outcomes and comorbidities were documented using the ICD‐9 codes, whilst mortality outcomes were recorded using the ICD‐10 coding system. ICD‐10 codes C00‐C97 were used to identify all‐cause cancer mortality. The ICD‐9 and ICD‐10 codes are summarised in Table S1.

Descriptive statistics were used to summarise baseline characteristics for this cohort. Mean and standard deviation (SD) was used to represent continuous variables, while a number and percentage were used to represent categorical variables. Propensity score matching with a 1:1 ratio between SGLT2I and DPP4I users and patients with and without new‐onset overall cancer risk based on demographics, prior comorbidities, laboratory data, medication usage, Charlson comorbidity index and abbreviated modification of diet in renal disease were performed using the nearest neighbour strategy with the Calliper set at 0.1. Univariable and multivariable Cox proportional regressions were performed for both before and after matching to identify significant predictors of new‐onset all‐cause cancer occurrence and mortality. This is further corroborated by the inverse probability of treatment weighting using propensity scores and calculating incidence rate ratios. Cumulative incidence curves were also calculated to visually depict the difference in the time‐to‐adverse event by comparing the SGLT2I and DPP4I groups. *p* < 0.05 was considered statistically significant. Statistical analyses and propensity score matching was performed with RStudio software (version: 1.1.456) and Stata software (version 13.0), respectively.

## 3 RESULTS

### 3.1 Baseline characteristics

**FIGURE 1:**
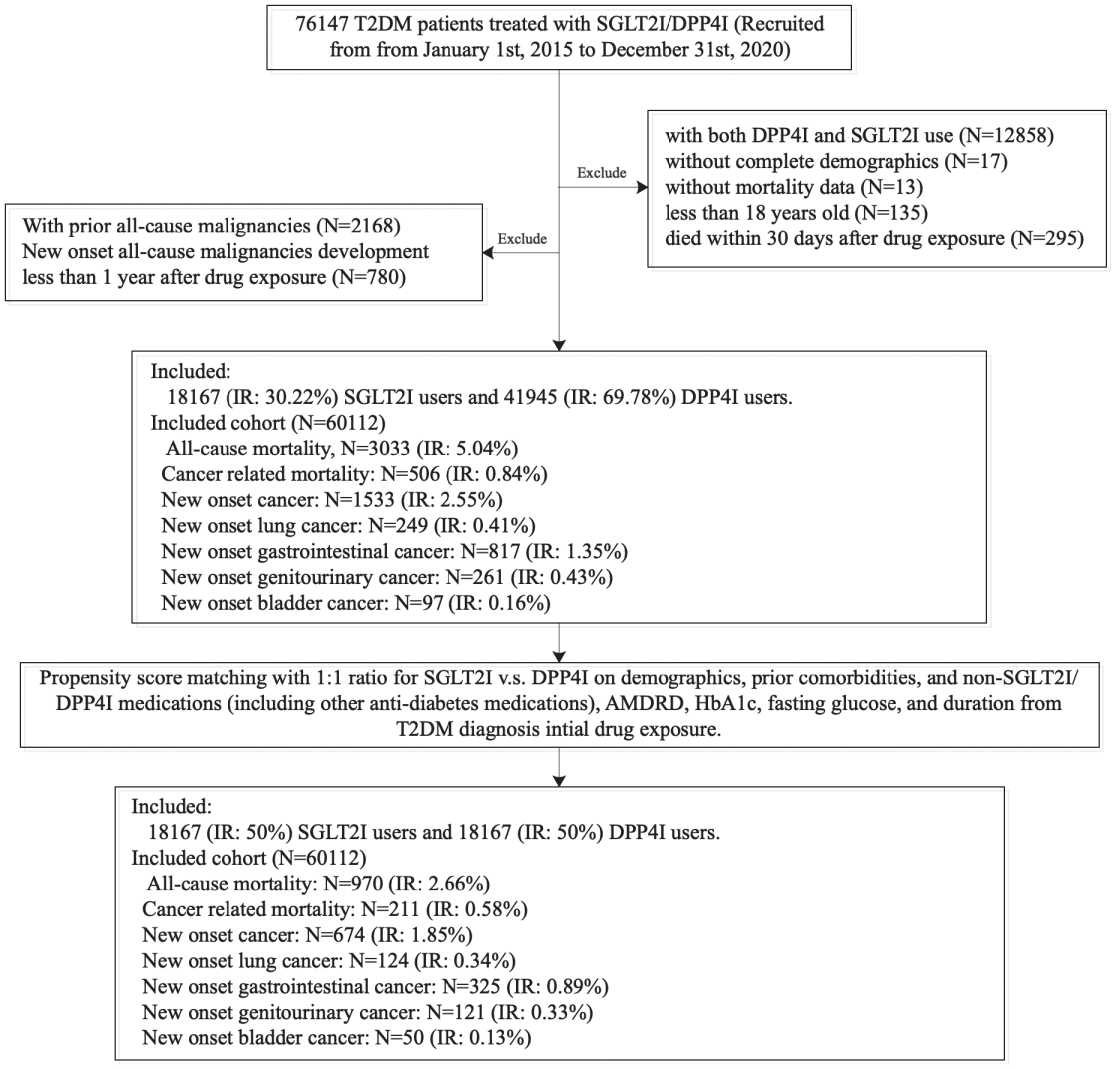
Procedures of data processing for the study cohort. DPP4I, dipeptidyl peptidase‐4 inhibitors; IR, incidence rate; SGLT2I, sodium‐glucose cotransporter‐2 inhibitors.

This study included 60,112 T2DM patients (mean baseline age: 62.1 ± 12.4 years, male: 56.36%, mean diabetes mellitus disease duration to baseline date: 640.6 ± 1264.0 days), of which 18,167 patients were SGLT2I users and 41,945 patients were DPP4I users. In the SGLT2I subgroup, the corresponding number of patients on individual SGLT2Is is as follows: 4523 (24.89%) on canagliflozin, 10,556 (58.10%) on dapagliflozin, 3780 (20.80%) on empagliflozin and 2527 (13.90%) on ertugliflozin. During the follow‐up period, 1533 patients developed new‐onset overall cancer, 3033 patients died from any cause, of which 506 patients died due to cancer‐related causes. Data on specific types of new‐onset overall cancers were also extracted: 249 patients developed new‐onset lung cancer, 817 patients developed new‐onset gastrointestinal cancer, 201 patients developed new‐onset breast cancer, 261 patients developed new‐onset genitourinary cancer and 97 patients developed new‐onset bladder cancer (Figure 1). The baseline characteristics for continuous and discrete variables of demographics, laboratory and medication histories for patients before and after matching are shown in Table 1, and Table S3A–C. The method of variability (standard deviation) calculation is shown in Table S2.

**TABLE 1:** Baseline and clinical characteristics of patients with SGLT2I versus DPP4I use before and after propensity score matching (1:1).

	Before matching				After matching			
Characteristics	All (*N* = 60,112) Mean (SD); *N* or count (%)	SGLT2I users (*N* = 18,167) Mean (SD); *N* or count (%)	DPP4I users (*N* = 41,945) Mean (SD); *N* or count (%)	SMD	All (*N* = 36,334) Mean (SD); *N* or count (%)	SGLT2I users (*N* = 18,167) Mean (SD); *N* or count (%)	DPP4I users (*N* = 18,167) Mean (SD); *N* or count (%)	SMD
Demographics								
Male gender	33,883 (56.36%)	11,138 (61.30%)	22,745 (54.22%)	0.14	22,288 (61.34%)	11,147 (61.35%)	11,141 (61.32%)	<0.01
Female gender	26,229 (43.63%)	7029 (38.69%)	19,200 (45.77%)	0.14	14,046 (38.65%)	7020 (38.64%)	7026 (38.67%)	<0.01
Baseline age, years	62.1 (12.4); *n* = 60,112	57.8 (11.2); *n* = 18,167	63.9 (12.5); *n* = 41,945	0.51^a^	58.2 (11.0); *n* = 36,334	57.8 (11.2); *n* = 18,167	58.7 (10.9); *n* = 18,167	0.08
18–50	9057 (15.06%)	3878 (21.34%)	5179 (12.34%)	0.24^a^	7212 (19.84%)	3888 (21.40%)	3324 (18.29%)	0.08
50–60	17,550 (29.19%)	6524 (35.91%)	11,026 (26.28%)	0.21^a^	13,285 (36.56%)	6543 (36.01%)	6742 (37.11%)	0.02
60–70	18,029 (29.99%)	5464 (30.07%)	12,565 (29.95%)	<0.01	11,194 (30.80%)	5457 (30.03%)	5737 (31.57%)	0.03
70–80	10,338 (17.19%)	1883 (10.36%)	8455 (20.15%)	0.27^a^	3766 (10.36%)	1870 (10.29%)	1896 (10.43%)	<0.01
>80	5145 (8.55%)	421 (2.31%)	4724 (11.26%)	0.36^a^	881 (2.42%)	412 (2.26%)	469 (2.58%)	0.02
Past comorbidities								
Charlson standard comorbidity index	1.9 (1.4); *n* = 60,112	1.5 (1.2); *n* = 18,167	2.1 (1.4); *n* = 41,945	0.45^a^	1.5 (1.2); *n* = 36,334	1.52 (1.17); *n* = 18,167	1.55 (1.14); *n* = 18,167	0.02
Duration from earliest diabetes mellitus date to baseline date, day	619.9 (1341.9); *n* = 60,112	616.9 (1352.3); *n* = 18,167	621.2 (1337.4); *n* = 41,945	<0.01	590.9 (1323.1); *n* = 36,334	618.7 (1354.2); *n* = 18,167	563.1 (1290.6); *n* = 18,167	0.04
Hypertension	13,376 (22.25%)	4284 (23.58%)	9092 (21.67%)	0.05	8325 (22.91%)	4283 (23.57%)	4042 (22.24%)	0.03
Hyperlipidaemia	1620 (2.69%)	66 2 (3.64%)	958 (2.28%)	0.08	1265 (3.48%)	659 (3.62%)	606 (3.33%)	0.02
Hypotension	350 (0.58%)	76 (0.41%)	274 (0.65%)	0.03	152 (0.41%)	75 (0.41%)	77 (0.42%)	<0.01
Overweight, obesity and hyperalimentation	432 (0.71%)	297 (1.63%)	135 (0.32%)	0.13	576 (1.58%)	300 (1.65%)	276 (1.51%)	0.01
Gout	1510 (2.51%)	394 (2.16%)	1116 (2.66%)	0.03	782 (2.15%)	394 (2.16%)	388 (2.13%)	<0.01
Heart failure	1629 (2.70%)	447 (2.46%)	1182 (2.81%)	0.02	874 (2.40%)	447 (2.46%)	427 (2.35%)	0.01
Acute myocardial infarction	1521 (2.53%)	616 (3.39%)	905 (2.15%)	0.08	1210 (3.33%)	613 (3.37%)	597 (3.28%)	<0.01
Ischaemic heart disease	5680 (9.44%)	2342 (12.89%)	3338 (7.95%)	0.16	4466 (12.29%)	2333 (12.84%)	2133 (11.74%)	0.03
Peripheral vascular disease	375 (0.62%)	97 (0.53%)	278 (0.66%)	0.02	194 (0.53%)	99 (0.54%)	95 (0.52%)	<0.01
Stroke/transient ischaemic attack	1777 (2.95%)	474 (2.60%)	1303 (3.10%)	0.03	940 (2.58%)	477 (2.62%)	463 (2.54%)	<0.01
Atrial fibrillation	1325 (2.20%)	388 (2.13%)	937 (2.23%)	0.01	766 (2.10%)	386 (2.12%)	380 (2.09%)	<0.01
Diabetic eye disease	4045 (6.72%)	1322 (7.27%)	2723 (6.49%)	0.03	2459 (6.76%)	1321 (7.27%)	1138 (6.26%)	0.04
Alcohol dependence	119 (0.19%)	22 (0.12%)	97 (0.23%)	0.03	44 (0.12%)	23 (0.12%)	21 (0.11%)	<0.01
Chronic liver disease and cirrhosis	1195 (1.98%)	513 (2.82%)	682 (1.62%)	0.08	995 (2.73%)	515 (2.83%)	480 (2.64%)	0.01
Viral hepatitis	629 (1.04%)	209 (1.15%)	420 (1.00%)	0.01	414 (1.13%)	211 (1.16%)	203 (1.11%)	<0.01
History of acute liver injury	159 (0.26%)	36 (0.19%)	123 (0.29%)	0.02	72 (0.19%)	36 (0.19%)	36 (0.19%)	<0.01
Other liver disease	612 (1.01%)	163 (0.89%)	449 (1.07%)	0.02	324 (0.89%)	160 (0.88%)	164 (0.90%)	<0.01
Autoimmune disease tissue	620 (1.03%)	198 (1.08%)	422 (1.00%)	0.01	386 (1.06%)	200 (1.10%)	186 (1.02%)	0.01
Infections by pathogens associated with cancer development (*Helicobacterpylori*, *Human papillomavirus*, *Infectious mononucleosis*)	13 (0.02%)	6 (0.03%)	7 (0.01%)	0.01	12 (0.03%)	6 (0.03%)	6 (0.03%)	<0.01
Chronic obstructive pulmonary disease	69 (0.11%)	12 (0.06%)	57 (0.13%)	0.02	24 (0.06%)	12 (0.06%)	12 (0.06%)	<0.01
Gastrointestinal disease	1353 (2.25%)	349 (1.92%)	1004 (2.39%)	0.03	690 (1.89%)	350 (1.92%)	340 (1.87%)	<0.01
Medications								
SGLT2I vs. DPP4I	18,167 (30.22%)	18,167 (100.00%)	0 (0.00%)	inf^a^	18,167 (50.00%)	18,167 (100.00%)	0 (0.00%)	inf^a^
SGLT2I frequency	7.4 (10.1); *n* = 18,167	7.4 (10.1); *n* = 18,167	–	–	7.3 (10.0); *n* = 18,167	7.3 (10.0); *n* = 18,167	–	–
DPP4I frequency	5.5 (7.2); *n* = 41,945	–	5.5 (7.2); *n* = 41,945	–	6.3 (8.0); *n* = 18,167	–	6.3 (8.0); *n* = 18,167	–
SGLT2I duration, days	538.1 (674.5); *n* = 18,167	538.1 (674.5); *n* = 18,167	–	–	538.5 (673.6); *n* = 18,167	538.5 (673.6); *n* = 18,167	–	–
DPP4I duration, days	528.9 (301.0); *n* = 41,945	–	528.9 (301.0); *n* = 41,945	–	575.3 (360.3); *n* = 18,167	–	575.3 (360.3); *n* = 18,167	–
Metformin	54,407 (90.50%)	16,917 (93.11%)	37,490 (89.37%)	0.13	33,945 (93.42%)	16,923 (93.15%)	17,022 (93.69%)	0.02
Sulphonylurea	46,521 (77.39%)	12,899 (71.00%)	33,622 (80.15%)	0.21^a^	26,376 (72.59%)	12,889 (70.94%)	13,487 (74.23%)	0.07
Insulin	29,349 (48.82%)	9517 (52.38%)	19,832 (47.28%)	0.1	19,074 (52.49%)	9509 (52.34%)	9565 (52.65%)	0.01
Acarbose	1566 (2.60%)	751 (4.13%)	815 (1.94%)	0.13	1429 (3.93%)	744 (4.09%)	685 (3.77%)	0.02
Thiazolidinedone	12,046 (20.03%)	5130 (28.23%)	6916 (16.48%)	0.28^a^	9569 (26.33%)	5134 (28.26%)	4435 (24.41%)	0.09
Glucagon‐like peptide‐1 receptor agonists	2044 (3.40%)	1505 (8.28%)	539 (1.28%)	0.33^a^	2797 (7.69%)	1506 (8.28%)	1291 (7.10%)	0.04
ACEI/ARB	18,249 (30.35%)	11,149 (61.36%)	7100 (16.92%)	1.02^a^	22,352 (61.51%)	11,152 (61.38%)	11,200 (61.65%)	0.01
Antidepressants	2742 (4.56%)	1665 (9.16%)	1077 (2.56%)	0.28^a^	2971 (8.17%)	1661 (9.14%)	1310 (7.21%)	0.07
Antihypertensive drugs	2311 (3.84%)	1681 (9.25%)	630 (1.50%)	0.35^a^	3051 (8.39%)	1680 (9.24%)	1371 (7.54%)	0.06
Antihepatitis	809 (1.34%)	336 (1.84%)	473 (1.12%)	0.06	664 (1.82%)	337 (1.85%)	327 (1.79%)	<0.01
Anticoagulants	29,401 (48.91%)	18,166 (99.99%)	11,235 (26.78%)	2.34^a^	36,332 (99.99%)	18,166 (99.99%)	18,166 (99.99%)	<0.01
Antiplatelets	10,115 (16.82%)	5981 (32.92%)	4134 (9.85%)	0.59^a^	11,686 (32.16%)	5975 (32.88%)	5711 (31.43%)	0.03
Statins and fibrates	34,307 (57.07%)	13,773 (75.81%)	20,534 (48.95%)	0.58^a^	27,749 (76.37%)	13,780 (75.85%)	13,969 (76.89%)	0.02
Nitrates	4652 (7.73%)	2688 (14.79%)	1964 (4.68%)	0.35^a^	5278 (14.52%)	2684 (14.77%)	2594 (14.27%)	0.01
Non‐steroidal anti‐inflammatory drugs	9719 (16.16%)	5734 (31.56%)	3985 (9.50%)	0.57^a^	11,369 (31.29%)	5730 (31.54%)	5639 (31.03%)	0.01
Diuretics	10,179 (16.93%)	5633 (31.00%)	4546 (10.83%)	0.51^a^	11,095 (30.53%)	5628 (30.97%)	5467 (30.09%)	0.02
Beta‐blockers	8075 (13.43%)	4696 (25.84%)	3379 (8.05%)	0.49^a^	9261 (25.48%)	4694 (25.83%)	4567 (25.13%)	0.02
Calcium channel blockers	14,154 (23.54%)	8107 (44.62%)	6047 (14.41%)	0.70^a^	16,462 (45.30%)	8102 (44.59%)	8360 (46.01%)	0.03
Subclinical biomarkers								
Abbreviated MDRD, mL/min/1.73 m^2^	81.4 (27.9); *n* = 49,633	90.1 (23.8); *n* = 15,520	77.4 (28.8); *n* = 34,113	0.48^a^	88.6 (23.5); *n* = 29,687	90.1 (23.8); *n* = 15,514	86.9 (23.1); *n* = 14,173	0.14
Most severe renal damage (<15 mL/min/1.73 m^2^)	436.0 (0.72%)	16.0 (0.08%)	420.0 (1.00%)	0.12	40.0 (0.11%)	16.0 (0.08%)	24.0 (0.13%)	0.01
Severe renal damage ([15, 30] mL/min/1.73 m^2^)	1108.0 (1.84%)	41.0 (0.22%)	1067.0 (2.54%)	0.2	111.0 (0.30%)	39.0 (0.21%)	72.0 (0.39%)	0.03
Moderate to severe renal damage ([30, 45] mL/min/1.73 m^2^)	3548.0 (5.90%)	265.0 (1.45%)	3283.0 (7.82%)	0.31^a^	657.0 (1.80%)	261.0 (1.43%)	396.0 (2.17%)	0.06
Mild to moderate renal damage ([45, 60] mL/min/1.73 m^2^)	5816.0 (9.67%)	999.0 (5.49%)	4817.0 (11.48%)	0.22^a^	2222.0 (6.11%)	1000.0 (5.50%)	1222.0 (6.72%)	0.05
Mild renal damage ([60, 90] mL/min/1.73 m^2^)	19920.0 (33.13%)	6788.0 (37.36%)	13132.0 (31.30%)	0.13	12997.0 (35.77%)	6783.0 (37.33%)	6214.0 (34.20%)	0.07
Chronic kidney disease (>90 mL/min/1.73 m^2^)	18805.0 (31.28%)	7411.0 (40.79%)	11394.0 (27.16%)	0.29^a^	13660.0 (37.59%)	7415.0 (40.81%)	6245.0 (34.37%)	0.13
Neutrophil‐to‐lymphocyte ratio	3.4 (4.5); *n* = 23,978	3.0 (3.9); *n* = 8133	3.6 (4.8); *n* = 15,845	0.15	3.1 (3.8); *n* = 15,970	3.0 (3.9); *n* = 8146	3.2 (3.7); *n* = 7824	0.05
Platelet‐to‐lymphocyte ratio	142.0 (148.2); *n* = 23,976	133.5 (168.7); *n* = 8131	146.4 (136.3); *n* = 15,845	0.08	133.6 (137.8); *n* = 15,969	133.3 (168.5); *n* = 8144	133.9 (95.8); *n* = 7825	<0.01
Neutrophil‐to‐high‐density lipoprotein ratio	0.3 (0.2); *n* = 21,968	0.27 (0.16); *n* = 7755	0.27 (0.2); *n* = 14,213	0.01	0.3 (0.2); *n* = 15,080	0.27 (0.16); *n* = 7770	0.27 (0.16); *n* = 7310	0.01
Low density lipoprotein ratio‐to‐high density lipoprotein ratio	2.1 (0.9); *n* = 46,116	2.14 (0.83); *n* = 14,654	2.1 (0.86); *n* = 31,462	0.05	2.1 (0.8); *n* = 27,933	2.14 (0.83); *n* = 14,653	2.13 (0.84); *n* = 13,280	0.01
Triglyceride‐glucose index	7.6 (0.7); *n* = 42,025	7.6 (0.7); *n* = 13,571	7.5 (0.7); *n* = 28,454	0.15	7.6 (0.7); *n* = 25,514	7.64 (0.72); *n* = 13,576	7.64 (0.73); *n* = 11,938	0.01
Protein‐to‐creatinine ratio	3.0 (1.5); *n* = 29,872	3.4 (1.5); *n* = 10,496	2.8 (1.5); *n* = 19,376	0.34^a^	3.3 (1.5); *n* = 19,746	3.4 (1.5); *n* = 10,502	3.2 (1.4); *n* = 9244	0.13
Aspartate aminotransferase‐to‐alanine transaminase ratio	1.1 (3.2); *n* = 8773	0.9 (1.0); *n* = 3284	1.2 (3.9); *n* = 5489	0.08	0.9 (0.8); *n* = 6377	0.92 (0.99); *n* = 3279	0.94 (0.5); *n* = 3098	0.03
Complete blood counts, renal and liver functions								
Mean corpuscular volume, fL	87.1 (7.5); *n* = 29,998	86.6 (7.2); *n* = 10,523	87.4 (7.6); *n* = 19,475	0.1	86.7 (7.2); *n* = 19,809	86.6 (7.2); *n* = 10,529	86.8 (7.3); *n* = 9280	0.03
Potassium, mmol/L	4.3 (0.5); *n* = 49,471	4.3 (0.4); *n* = 15,489	4.4 (0.5); *n* = 33,982	0.1	4.3 (0.4); *n* = 29,604	4.31 (0.43); *n* = 15,483	4.27 (0.45); *n* = 14,121	0.1
Albumin, g/L	41.9 (3.8); *n* = 37,720	42.5 (3.3); *n* = 13,085	41.5 (4.0); *n* = 24,635	0.27^a^	42.5 (3.3); *n* = 24,595	42.5 (3.3); *n* = 13,089	42.4 (3.4); *n* = 11,506	0.03
Sodium, mmol/L	139.3 (2.9); *n* = 49,494	139.1 (2.7); *n* = 15,492	139.3 (3.0); *n* = 34,002	0.07	139.3 (2.7); *n* = 29,612	139.1 (2.7); *n* = 15,486	139.4 (2.7); *n* = 14,126	0.08
Urea, mmol/L	6.4 (3.3); *n* = 49,483	5.7 (2.0); *n* = 15,486	6.8 (3.7); *n* = 33,997	0.34^a^	5.8 (2.1); *n* = 29,607	5.7 (2.0); *n* = 15,480	5.8 (2.2); *n* = 14,127	0.05
Protein, g/L	73.9 (5.4); *n* = 35,490	74.3 (4.9); *n* = 12,386	73.8 (5.6); *n* = 23,104	0.1	74.4 (5.0); *n* = 23,401	74.3 (4.9); *n* = 12,387	74.5 (5.0); *n* = 11,014	0.05
Creatinine, μmol/L	92.5 (71.5); *n* = 49,633	79.0 (28.9); *n* = 15,520	98.6 (83.2); *n* = 34,113	0.31^a^	80.4 (33.0); *n* = 29,687	79.0 (28.9); *n* = 15,514	82.0 (36.9); *n* = 14,173	0.09
Alkaline phosphatase, U/L	76.2 (30.1); *n* = 37,836	73.5 (26.1); *n* = 13,091	77.6 (31.9); *n* = 24,745	0.14	74.8 (27.2); *n* = 24,597	73.5 (26.1); *n* = 13,095	76.2 (28.3); *n* = 11,502	0.1
Aspartate transaminase, U/L	28.2 (53.4); *n* = 15,006	28.1 (26.9); *n* = 5161	28.2 (63.0); *n* = 9845	<0.01	28.8 (30.5); *n* = 10,477	28.2 (26.9); *n* = 5162	29.4 (33.6); *n* = 5315	0.04
Alanine transaminase, U/L	29.4 (33.6); *n* = 32,037	32.1 (28.1); *n* = 11,145	28.0 (36.1); *n* = 20,892	0.13	32.3 (27.5); *n* = 20,438	32.1 (28.2); *n* = 11,145	32.4 (26.6); *n* = 9293	0.01
Bilirubin, μmol/L	11.3 (6.7); *n* = 37,645	11.4 (5.6); *n* = 13,060	11.2 (7.1); *n* = 24,585	0.04	11.4 (5.7); *n* = 24,545	11.4 (5.6); *n* = 13,064	11.3 (5.8); *n* = 11,481	0.01
Lipid profiles								
Triglyceride, mmol/L	1.7 (1.6); *n* = 46,943	1.8 (1.7); *n* = 14,910	1.7 (1.5); *n* = 32,033	0.06	1.8 (1.8); *n* = 28,423	1.8 (1.75); *n* = 14,910	1.82 (1.84); *n* = 13,513	0.01
Low‐density lipoprotein, mmol/L	2.4 (0.8); *n* = 46,121	2.37 (0.8); *n* = 14,658	2.4 (0.8); *n* = 31,463	0.04	2.4 (0.8); *n* = 27,937	2.37 (0.8); *n* = 14,657	2.39 (0.79); *n* = 13,280	0.03
High‐density lipoprotein, mmol/L	1.2 (0.3); *n* = 46,872	1.16 (0.31); *n* = 14,885	1.21 (0.33); *n* = 31,987	0.15	1.2 (0.3); *n* = 28,370	1.16 (0.31); *n* = 14,885	1.18 (0.32); *n* = 13,485	0.06
Total cholesterol, mmol/L	4.3 (1.0); *n* = 46,986	4.3 (1.0); *n* = 14,929	4.4 (1.0); *n* = 32,057	0.04	4.3 (1.0); *n* = 28,445	4.3 (1.0); *n* = 14,929	4.4 (1.0); *n* = 13,516	0.05
Haemoglobin A1C, %	8.1 (1.5); *n* = 48,920	8.3 (1.6); *n* = 15,353	8.0 (1.5); *n* = 33,567	0.21^a^	8.3 (1.6); *n* = 29,345	8.3 (1.57); *n* = 15,352	8.26 (1.61); *n* = 13,993	0.03
Fasting glucose, mmol/L	9.9 (3.3); *n* = 34,323	10.1 (3.2); *n* = 11,828	9.8 (3.4); *n* = 22,495	0.08	10.1 (3.3); *n* = 22,141	10.1 (3.2); *n* = 11,841	10.0 (3.5); *n* = 10,300	0.02

Abbreviations: ACEI/ARB, Angiotensin‐converting enzyme inhibitors Angiotensin receptor blockers; DPP4I, dipeptidyl peptidase‐4 inhibitor; MDRD, modification of diet in renal disease; SD, standard deviation; SGLT2I, sodium glucose cotransporter‐2 inhibitor.

^a^ For SMD ≥0.2.

**FIGURE 2:**
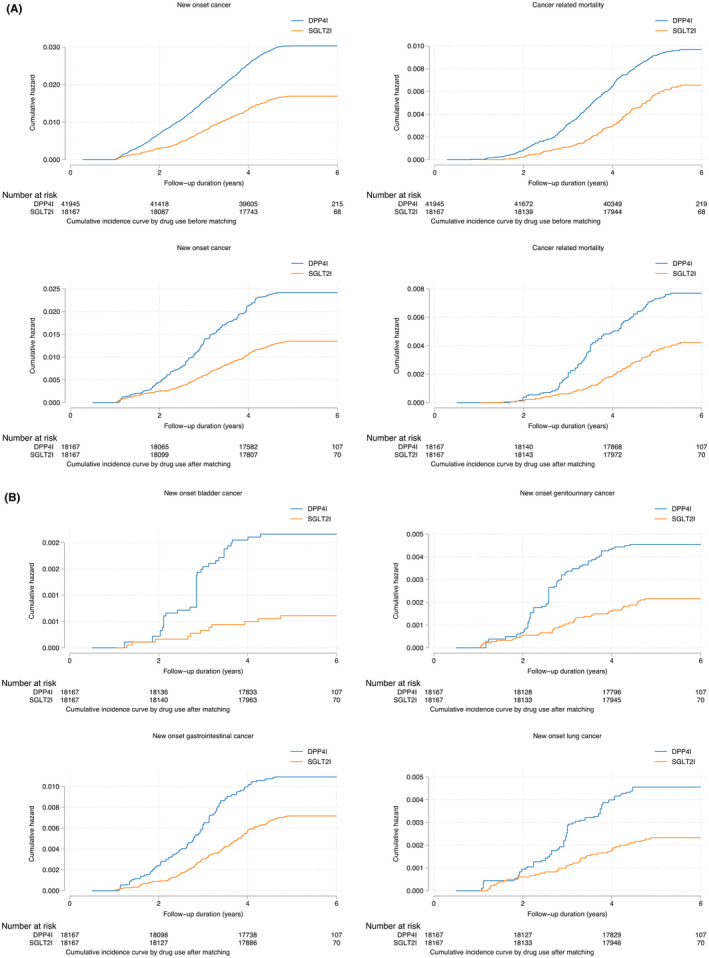
(A) Cumulative incidence curves for new‐onset cancer and cancer‐related mortality stratified by drug exposure effects of SGLT2I and DPP4I before and after propensity score matching (1:1). (B) Cumulative incidence curves for different new‐onset cancer outcomes stratified by drug exposure effects of SGLT2I and DPP4I in the matched cohort.

**FIGURE 3:**
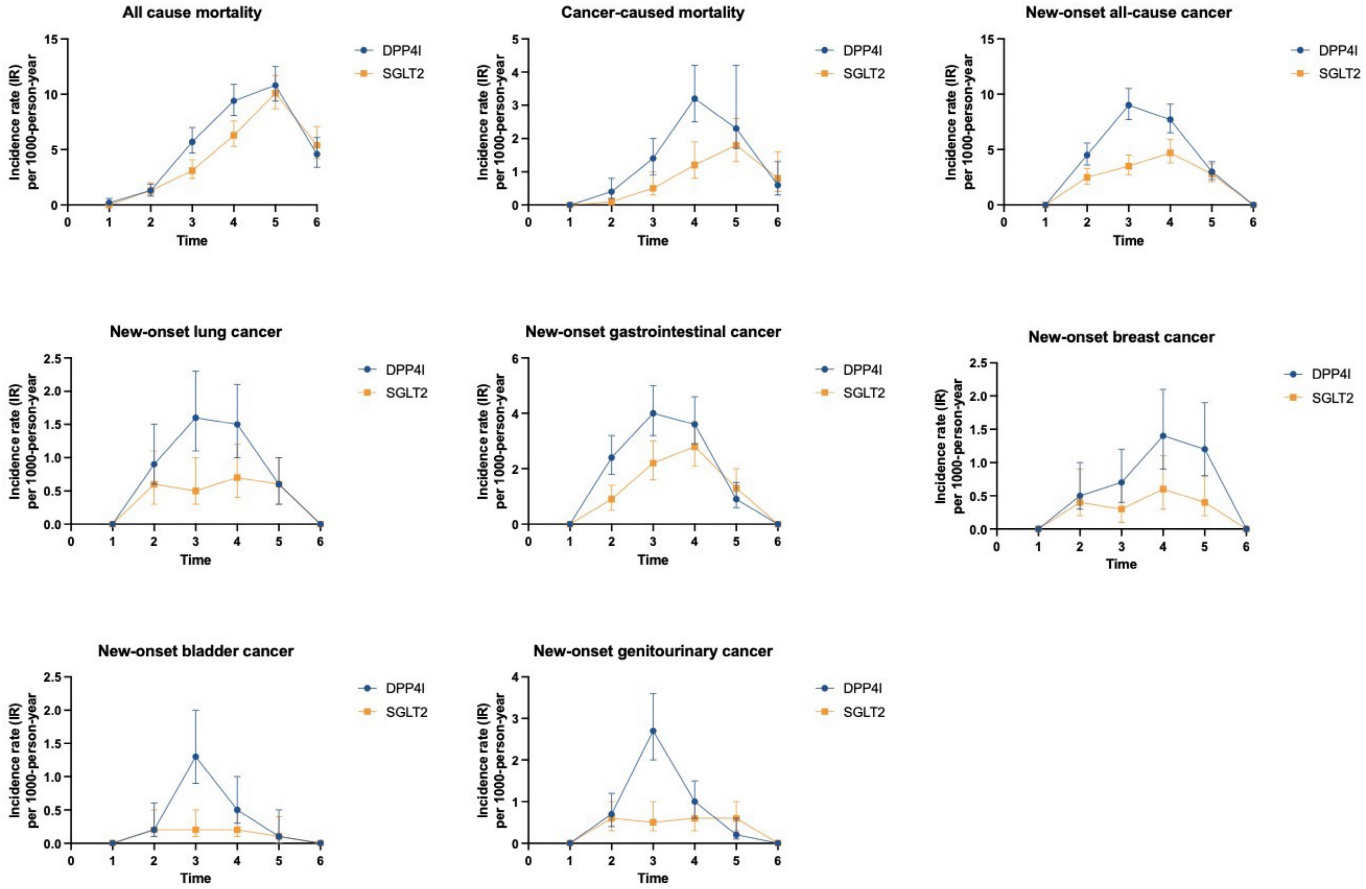
Summary figures of comparing annual incidence ratios with 95% CIs of different adverse events stratified by drug use.

The cumulative incidence of primary and secondary outcomes after propensity score matching is shown in Table 2A. The cumulative incidences of these outcomes stratified by initial drug exposure age, drug use, the combination of gender and drug exposure and combination of age and drug exposure effects are summarised by cumulative incidence curves (Figures 2B). Gender‐based and age‐based trends in the incidence of the different outcomes are shown in Figure S3A,B. Furthermore, summary figures of comparing annual incidence ratios with 95% CIs of different adverse events stratified by drug use are presented in Figure 3A.

**TABLE 2A:** Annualised incidence rate (IR) per 1000 person‐years of primary and secondary cancer outcomes, all‐cause mortality and cancer related mortality in the cohort before and after 1:1 propensity score matching.

Before matching				After 1:1 propensity score matching			
All‐cause mortality							
Overall	Person‐year	Events	IR [95% CI]	Overall	Person‐year	Events	IR [95% CI]
	3.29 × 10^5^	3033	9.2 [8.6–9.6]		2.01 × 10^5^	970	4.8 [4.5–5.1]
Cancer‐related mortality							
Overall	Person‐year	Events	IR [95% CI]	Overall	Person‐year	Events	IR [95% CI]
	3.29 × 10^5^	506	1.5 [1.4–1.7]		2.01 × 10^5^	211	1.1 [0.9–1.2]
New‐onset all‐cause cancer							
Overall	Person‐year	Events	IR [95% CI]	Overall	Person‐year	Events	IR [95% CI]
	3.26 × 10^5^	1533	4.7 [4.5–4.9]		2.00 × 10^5^	674	3.4 [3.1–3.6]
New‐onset lung cancer							
Overall	Person‐year	Events	IR [95% CI]	Overall	Person‐year	Events	IR [95% CI]
	3.28 × 10^5^	249	0.8 [0.7–0.9]		2.01 × 10^5^	124	0.6 [0.5–0.7]
New‐onset gastrointestinal cancer							
Overall	Person‐year	Events	IR [95% CI]	Overall	Person‐year	Events	IR [95% CI]
	3.27 × 10^5^	817	2.5 [2.3–2.7]		2.00 × 10^5^	325	1.6 [1.5–1.8]
New‐onset bladder cancer							
Overall	Person‐year	Events	IR [95% CI]	Overall	Person‐year	Events	IR [95% CI]
	3.29 × 10^5^	97	0.3 [0.2–0.4]		2.01 × 10^5^	50	0.2 [0.2–0.3]
New‐onset genitourinary cancer							
Overall	Person‐year	Events	IR [95% CI]	Overall	Person‐year	Events	IR [95% CI]
	3.28 × 10^5^	261	0.8 [0.7–0.9]		2.01 × 10^5^	121	0.6 [0.5–0.7]

### 3.2 Cox regression

The results of univariable Cox regression analysis for predicting general and pre‐specified cancer risk are displayed in Table S4A,B. Significant variables in univariable regression were subsequently incorporated into multivariable models to evaluate the relationship between SGLT2I and DPP4I with malignancy. As shown in Table 2B, after adjustment for significant demographics, past comorbidities, non‐SGLT2I/DPP4I medications, abbreviated MDRD, fasting glucose, HbA1c and duration from earliest diabetes mellitus date to initial drug exposure date, SGLT2I were associated with a comparatively decreased risk of all‐cause mortality (HR: 0.92; 95% CI: 0.84–0.99; *p* = 0.04), cancer‐related mortality (HR: 0.58; 95% CI: 0.42–0.80; *p*  ≤ 0.001), as well as a 30% reduction in the risk of new‐onset overall cancer (HR: 0.70; 95% CI: 0.59–0.84; *p* = <0.001). When stratified by cancer subtype, SGLT2I were related to a lower risk of new‐onset breast cancer (HR: 0.51; 95% CI: 0.32–0.80; *p* = <0.001), but not with other malignancies. With subgroup analysis comparing DPP4I to different subtypes of SGLT2I, dapagliflozin (HR: 0.78; 95% CI: 0.64–0.95; *p* = 0.01) and ertugliflozin (HR: 0.65; 95% CI: 0.43–0.98; *p* = 0.04) both demonstrated superiority in relation to new‐onset overall cancer development, with the former also presenting with a relatively lower risk of breast cancer (HR: 0.48; 95% CI: 0.27–0.83; *p* = 0.001). There were no observable differences when comparing the use of either canagliflozin or empagliflozin with DPP4I in terms of overall or specific cancer risk.

**TABLE 2B:** Multivariable Cox regression models with adjustments to predict new all‐cause cancers in the matched cohort.

Characteristics	All‐cause mortality HR [95% CI]; *p* value	Cancer‐related mortality HR [95% CI]; *p* value	New‐onset cancer HR [95% CI]; *p* value	New‐onset lung cancer HR [95% CI]; *p* value	New‐onset gastrointestinal cancer HR [95% CI]; *p* value	New‐onset breast cancer HR [95% CI]; *p* value	New‐onset genitourinary cancer HR [95% CI]; *p* value	New‐onset bladder cancer HR [95% CI]; *p* value
Model 1								
SGLT2I vs. DPP4I	0.84 [0.74–0.96]; 0.0085**	0.57 [0.43–0.75]; 0.0001***	0.58 [0.49–0.67]; <0.0001***	0.53 [0.37–0.77]; 0.0009***	0.68 [0.55–0.85]; 0.0008***	0.47 [0.31–0.71]; 0.0004***	0.48 [0.33–0.70]; 0.0002***	0.29 [0.15–0.56]; 0.0002***
Dapagliflozin vs. DPP4I	0.89 [0.77–1.03]; 0.1144	0.65 [0.46–0.91]; 0.0122*	0.64 [0.53–0.77]; <0.0001***	0.69 [0.45–1.07]; 0.0952	0.65 [0.49–0.85]; 0.0016**	0.42 [0.24–0.72]; 0.0018**	0.71 [0.46–1.09]; 0.1215	0.41 [0.19–0.92]; 0.0302*
Empagliflozin vs. DPP4I	0.75 [0.59–0.94]; 0.0143*	0.53 [0.30–0.96]; 0.0346*	0.75 [0.56–0.99]; 0.0423*	0.59 [0.29–1.21]; 0.1512	0.86 [0.59–1.26]; 0.4304	0.68 [0.31–1.46]; 0.3180	0.70 [0.35–1.37]; 0.2966	0.53 [0.17–1.72]; 0.2933
Canagliflozin vs. DPP4I	0.94 [0.77–1.14]; 0.5218	0.82 [0.53–1.28]; 0.3897	0.72 [0.55–0.93]; 0.0123*	0.47 [0.23–0.97]; 0.0406*	0.90 [0.64–1.27]; 0.5531	1.14 [0.63–2.03]; 0.6683	0.30 [0.12–0.74]; 0.0092**	0.14 [0.02–0.99]; 0.0485*
Ertugliflozin vs. DPP4I	1.08 [0.85–1.37]; 0.5497	0.80 [0.45–1.44]; 0.4605	0.58 [0.40–0.84]; 0.0044**	0.55 [0.22–1.35]; 0.1901	0.78 [0.48–1.25]; 0.2973	0.44 [0.14–1.40]; 0.1665	0.34 [0.11–1.07]; 0.0656	0.26 [0.04–1.88]; 0.1827
Model 2								
SGLT2I vs. DPP4I	0.83 [0.73–0.94]; 0.0033**	0.56 [0.42–0.74]; 0.0001***	0.57 [0.49–0.67]; <0.0001***	0.52 [0.36–0.76]; 0.0007***	0.68 [0.54–0.84]; 0.0006***	0.48 [0.31–0.72]; 0.0005***	0.48 [0.33–0.70]; 0.0001***	0.28 [0.14–0.55]; 0.0002***
Dapagliflozin vs. DPP4I	0.87 [0.75–1.01]; 0.0677	0.64 [0.46–0.90]; 0.0102*	0.63 [0.52–0.77]; <0.0001***	0.68 [0.44–1.05]; 0.0833	0.64 [0.49–0.84]; 0.0013**	0.42 [0.24–0.73]; 0.0020**	0.71 [0.46–1.09]; 0.1163	0.41 [0.18–0.91]; 0.0294*
Empagliflozin vs. DPP4I	0.75 [0.59–0.95]; 0.0169*	0.53 [0.30–0.95]; 0.0322*	0.74 [0.56–0.99]; 0.0392*	0.59 [0.29–1.20]; 0.1469	0.86 [0.58–1.25]; 0.4232	0.68 [0.31–1.46]; 0.3193	0.70 [0.35–1.37]; 0.2949	0.54 [0.17–1.72]; 0.2946
Canagliflozin vs. DPP4I	0.93 [0.76–1.13]; 0.4572	0.82 [0.53–1.27]; 0.3762	0.71 [0.55–0.93]; 0.0111*	0.47 [0.23–0.96]; 0.0380*	0.90 [0.64–1.26]; 0.5387	1.14 [0.64–2.05]; 0.6495	0.30 [0.12–0.74]; 0.0087**	0.13 [0.02–0.97]; 0.0465*
Ertugliflozin vs. DPP4I	1.06 [0.83–1.34]; 0.6492	0.79 [0.44–1.42]; 0.4313	0.57 [0.39–0.84]; 0.0040**	0.54 [0.22–1.33]; 0.1802	0.77 [0.48–1.24]; 0.2791	0.45 [0.14–1.42]; 0.1733	0.34 [0.11–1.07]; 0.0650	0.26 [0.04–1.88]; 0.1826
Model 3								
SGLT2I vs. DPP4I	0.89 [0.79–0.98]; 0.0440*	0.58 [0.44–0.77]; 0.0002***	0.59 [0.51–0.69]; <0.0001***	0.54 [0.37–0.79]; 0.0014**	0.71 [0.57–0.89]; 0.0028**	0.48 [0.31–0.73]; 0.0006***	0.49 [0.33–0.72]; 0.0002***	0.29 [0.15–0.56]; 0.0003***
Dapagliflozin vs. DPP4I	0.93 [0.80–1.07]; 0.3196	0.67 [0.48–0.94]; 0.0205*	0.66 [0.54–0.79]; <0.0001***	0.71 [0.46–1.10]; 0.1267	0.67 [0.51–0.88]; 0.0044**	0.43 [0.25–0.74]; 0.0024**	0.72 [0.47–1.11]; 0.1354	0.42 [0.19–0.93]; 0.0319*
Empagliflozin vs. DPP4I	0.77 [0.61–0.98]; 0.0316*	0.53 [0.30–0.95]; 0.0334*	0.75 [0.57–0.99]; 0.0436*	0.60 [0.29–1.22]; 0.1564	0.88 [0.60–1.29]; 0.5186	0.66 [0.31–1.43]; 0.2935	0.70 [0.36–1.39]; 0.3086	0.52 [0.16–1.68]; 0.2764
Canagliflozin vs. DPP4I	0.96 [0.79–1.17]; 0.6902	0.83 [0.54–1.29]; 0.4157	0.72 [0.55–0.93]; 0.0132*	0.47 [0.23–0.96]; 0.0384*	0.91 [0.65–1.29]; 0.6062	1.11 [0.62–2.00]; 0.7155	0.31 [0.13–0.75]; 0.0097**	0.14 [0.02–0.99]; 0.0486*
Ertugliflozin vs. DPP4I	1.13 [0.89–1.44]; 0.2992	0.83 [0.46–1.48]; 0.5242	0.59 [0.40–0.86]; 0.0060**	0.54 [0.22–1.32]; 0.1770	0.81 [0.50–1.30]; 0.3796	0.42 [0.13–1.32]; 0.1379	0.36 [0.11–1.12]; 0.0784	0.27 [0.04–1.93]; 0.1898
Model 4								
SGLT2I vs. DPP4I	0.92 [0.84–0.999]; 0.0419*	0.58 [0.42–0.80]; 0.0008***	0.70 [0.59–0.84]; 0.0001***	0.73 [0.47–1.13]; 0.1610	0.79 [0.62–1.01]; 0.0570	0.51 [0.32–0.80]; 0.0034**	0.70 [0.46–1.08]; 0.1037	0.55 [0.26–1.14]; 0.1075
Dapagliflozin vs. DPP4I	1.02 [0.87–1.19]; 0.8491	0.72 [0.50–1.04]; 0.0775	0.78 [0.64–0.95]; 0.0136*	0.91 [0.56–1.49]; 0.7141	0.76 [0.58–1.01]; 0.0631	0.48 [0.27–0.83]; 0.0095**	1.02 [0.64–1.62]; 0.9387	0.77 [0.33–1.78]; 0.5352
Empagliflozin vs. DPP4I	0.72 [0.54–0.94]; 0.0174*	0.46 [0.23–0.94]; 0.0323*	0.88 [0.65–1.19]; 0.4045	0.83 [0.38–1.79]; 0.6293	1.01 [0.68–1.50]; 0.9800	0.73 [0.32–1.67]; 0.4542	0.79 [0.37–1.72]; 0.5565	0.95 [0.29–3.11]; 0.9271
Canagliflozin vs. DPP4I	1.01 [0.81–1.24]; 0.9582	0.89 [0.55–1.44]; 0.6435	0.84 [0.64–1.10]; 0.2098	0.59 [0.27–1.29]; 0.1867	1.00 [0.70–1.43]; 0.9893	1.28 [0.69–2.36]; 0.4329	0.42 [0.17–1.03]; 0.0581	0.21 [0.03–1.56]; 0.1282
Ertugliflozin vs. DPP4I	1.19 [0.91–1.55]; 0.2021	0.70 [0.34–1.43]; 0.3276	0.65 [0.43–0.98]; 0.0375*	0.64 [0.23–1.75]; 0.3819	0.79 [0.47–1.33]; 0.3789	0.56 [0.18–1.77]; 0.3221	0.50 [0.16–1.59]; 0.2396	0.44 [0.06–3.25]; 0.4239

*Note*: Model 1 adjusted for significant demographics. Model 2 adjusted for significant demographics and past comorbidities. Model 3 adjusted for significant demographics, past comorbidities and non‐SGLT2I/DPP4I medications. Model 3 adjusted for significant demographics, past comorbidities and non‐SGLT2I/DPP4I medications. Model 4 adjusted for significant demographics, past comorbidities, non‐SGLT2I/DPP4I medications, abbreviated MDRD, fasting glucose, HbA1c and duration from earliest diabetes mellitus date to initial drug exposure date.

Abbreviations: CI, confidence interval; DPP4I, dipeptidyl peptidase‐4 inhibitor; HR, hazard ratio; SGLT2I: sodium glucose cotransporter‐2 inhibitor.

* *p* ≤ 0.05

** *p* ≤ 0.01

*** *p* ≤ 0.001.

### 3.3 Sensitivity analysis

To assess the predictivity of the models, sensitivity analysis was conducted to evaluate the effect of matching on the results, namely with inverse probability of treatment weighting (Table S5). The findings confirmed those of univariable cox regression, that SGLT2I administration was still associated with a lower risk of all‐cause mortality, cancer‐related mortality, new‐onset overall cancer as well as all pre‐specified cancers (lung, breast, gastrointestinal, genitourinary and bladder) when compared to DPP4I usage.

## 4 DISCUSSION

To the best of our knowledge, this is the first territory‐wide study that does a direct comparison of the effect of SGLT2I and DPP4I on overall and pre‐specified cancer risk in a cohort of Asian patients. The main findings of this study are as follows: In comparison with DPP4I, (i) SGLT2I were associated with a lower risk of all‐cause mortality, cancer‐related mortality and new‐onset overall cancer; (ii) SGLT2I were related to a lower risk of new‐onset breast cancer; (iii) when stratified according to the medication subtype, dapagliflozin and ertugliflozin both demonstrated a reduced risk of new‐onset malignancy, with the former also presenting with a lower risk of breast cancer.

Anti‐diabetic medications are amongst the most commonly prescribed drugs in the world, with the indications of some expanding beyond T2DM to other non‐diabetic cardiovascular and chronic kidney conditions.^21^, ^22^ The clinical practicality of these medications, coupled with their multifaceted systemic effects, warrants a thorough assessment of the safety of their long‐term usage, which has raised some important concerns in recent years. This is of specific importance concerning the comparatively newer classes of oral hypoglycaemic drugs, namely DPP4I and SGLT2I, the first of which were marketed in 2006 (Sitagliptin) and 2013 (Canagliflozin), respectively.^23^ Given the chronicity with which these medications are taken, a particularly significant outcome that is evaluated, unsurprisingly, is a cancer risk.

The majority of the comparative studies available in the existing literature have evaluated cancer risk across a wide range of anti‐diabetic drugs. Liu et al. performed a retrospective case‐controlled prognostic assessment for different anti‐diabetic medications, including metformin, thiazolidinediones, sulfonylureas, meglitinides, acarbose as well as insulin and its analogues, in turn revealing that apart from pioglitazone and insulin, the other therapies failed to show an association with cancer incidence. This relationship was maintained when stratifying outcomes by cancer type, namely for pancreatic, liver and lung cancer.^24^ In addition to this, certain investigations have demonstrated the protective effect of some of the older classes against cancer, most notably with metformin, which has demonstrated either a reduced association with cancer^25^, ^26^ or a lower incidence of cancer on follow‐up relative to other anti‐diabetic medications.^27^ Dąbrowski demonstrated that while some anti‐diabetic medications such as metformin and thiazolidinediones showed beneficial effects, the mitogenic effect of insulin could pose a harmful effect.^28^ Interestingly, short‐term insulin use was found to be associated with increased risk of cancer but not for a longer duration use. Amongst diabetic patients, long‐term usage of oral diabetic medication correlated with reduced pancreatic cancer risk.^29^ Incretin drugs and GLP‐1 receptor agonists supported a neutral association with cancer risk, with minimal preliminary evidence of its effect against various cancer types.^30^

Despite this, it should be noted that there is much more uncertainty about the malignancy risk of the somewhat newer anti‐diabetic medications. Regarding DPP4I, a meta‐analysis compiled by Zhao et al. did not report any association between these medications and malignancy, even when stratified by different subtypes of DPP4I.^12^ Similarly, the findings of another meta‐analysis lend further credence to this notion by not only failing to show a relationship with malignancy development but also purporting a potential protective effect of DPP4I against colorectal cancer.^31^ Preliminary evidence suggests that DPP4I can alter our immune system through the activation of cytokines, reduction of cellular growth factors and systemic inflammatory responses. Suppression of the catalytic activity of chemokines stimulated by DPP4 can thereby inhibit tumour cell proliferation. In a pilot study, patients with colorectal cancer who took DPP4I and had improved cancer prognosis showed changes in post‐operative lymphocyte count, platelet count, prognostic nutritional index, neutrophil‐to‐lymphocyte ratio and platelet‐to‐lymphocyte ratio.^32^ However, although these results may reflect much of the current school of thought concerning DPP4I, there have been some recent investigations that have suggested the possible existence of either a dose‐dependent or cancer‐type‐dependent correlation. As to the former, Chou et al. presented a higher incidence of colorectal cancer in patients on DPP4I who were receiving a high cumulative daily dose, but a corresponding lower risk of colorectal cancer amongst low cumulative dose users.^33^ As it pertains to the latter, there is evidence to suggest that whilst a relationship between DPP4I and overall cancer risk may not exist, these drugs are associated with specific cancer types when categorised, namely bladder, kidney and liver cancer as well as melanoma.^11^

Likewise, very much akin to that of DPP4I, the data centred around SGLT2I are also controversial. Most recently, a meta‐analysis performed by Benedetti et al. proposed a reduced cancer risk of SGLT2I when compared to placebo, with particular efficacy for dapagliflozin and ertugliflozin.^34^ These results are in line with that of the present study, which also demonstrated the superiority of dapagliflozin and ertugliflozin in relation to cancer risk. Such findings are further emphasised by that of Pelletier et al, which also failed to display an increased cancer risk with SGLT2I users, regardless of cancer type.^35^ Some researchers have identified regulatory functions of dapagliflozin on cell cycle and apoptosis, including an effect on reduced glucose uptake in CaKi‐1 cells.^36^ Specifically, studies have identified that the drugs attenuate cancer cell proliferation through changing the mitochondrial membrane potential and various membrane transporters such as the sodium and glucose cotransporter.^37^ Subsequently, the use of SGLT2I can reduce the viability and malignancy of carcinoma cells. The inhibitory effects of SGLT2I on glycolytic metabolism, cell cycle and intracellular ATP production in cancer cells are further supported in other research studies.^38^, ^39^, ^40^, ^41^, ^42^, ^43^ Alternatively, some animal studies demonstrate that dapagliflozin targets the reduction in glutathione metabolism, expression of pro‐inflammatory markers and the reversal of hyperinsulinemia to slow down tumour growth.^44^, ^45^, ^46^ However, the obscurity in the findings concerning SGLT2I primarily resides in the fact that the malignancy risk varies depending on the SGLT2I and cancer subtypes. One study commented the overexpression of SGLT1 and SGLT2 on lung, colorectal, head, ovarian, oral and neck carcinomas, supporting the therapeutic approach of using SGLT2Is for early tumour detection. However, current findings in this research field require further verification as non‐specific SGLT antibodies were used.^47^ Tang et al. showed that although the overall cancer incidence is lower with SGLT2I relative to other comparator drugs when analysing pre‐specified cancers, empagliflozin demonstrated a higher risk of bladder cancer whilst canagliflozin exhibited protective effects against gastrointestinal cancers.^10^ The ambiguity regarding SGLT2I is further compounded by other contrarian evidence suggesting a reduced risk of malignancy with empagliflozin relative to other oral hypoglycaemic agents, but instead, an increased risk when compared to placebo.^48^

Given the relatively newer status of DPP4I and SGLT2I, there is a paucity of literature comparing the non‐diabetic outcomes associated with these medications. Au et al. showcased a reduced incidence of pneumonia and pneumonia‐related mortality with SGLT2I relative to DPP4I in patients from Hong Kong.^49^ In a Taiwanese cohort, SGLT2I similarly exhibited superiority in the risk of gout development when compared to DPP4I.^13^ Moreover, one retrospective study in a Taiwanese cohort demonstrated that SGLT2I usage was associated with a lower risk of cancer‐related mortality compared to DPP4I, akin to the results to our investigations.^12^ To date, in addition to our study, there is only one other that has directly assessed these two classes of medication and their respective cancer risks. The findings from this study indicated that the risk of a urinary tract and haematological malignancy with SGLT2I was half that of with DPP4I, albeit there were no other differences amongst other cancer subtypes.^14^ These findings are supported by that of this study, which has likewise showcased that the use of SGLT2I is associated with a 30% reduction in new‐onset overall cancer risk in comparison with DPP4I, though there were no observable differences in genitourinary or bladder malignancy development between the two drug classes.

### 4.1 Limitations

There are certain limitations present in this population‐based study. First, due to the observational nature of this study, acquired results may be susceptible to information bias due to missing data, coding errors or under coding. Second, the retrospective nature of the study suggests that all derived findings regarding the relationship between SGLT2I, DPP4I and new‐onset overall cancer were correlational in nature. Third, information on drug exposure could not be directly obtained, and was instead determined indirectly through prescription refills, which may pose a liability concern. Fourth, as the drug exposure duration could not be standardised, this may have influenced the primary and secondary outcomes of the study. Finally, due to the lack of codes in CDARS, information regarding medical history, such as smoking status, were unattainable and could have been a confounding variable to cancer risk.

### 4.2 Conclusions

SGLT2I use was associated with lower risks of all‐cause mortality, cancer‐related mortality and new‐onset overall cancer compared to DPP4I use after propensity score matching and multivariable adjustment.

## AUTHOR CONTRIBUTIONS

**Cheuk To Chung:** Conceptualization (equal); writing – original draft (equal); writing – review and editing (equal). **Oscar Hou In Chou:** Methodology (equal); validation (equal); visualization (equal). **Teddy Tai Loy Lee:** Investigation (equal); resources (equal); writing – review and editing (equal). **Edward Christopher Dee:** Conceptualization (equal); data curation (equal). **Kenrick Ng:** Resources (equal). **Wing Tak Wong:** Supervision (equal). **Tong Liu:** Investigation (equal); supervision (equal). **Sharen Lee:** Supervision (equal); writing – review and editing (equal). **Bernard Cheung:** Investigation (equal); supervision (equal). **Qingpeng Zhang:** Resources (equal). **Jiandong Zhou:** Formal analysis (equal); investigation (equal); methodology (equal); software (equal); visualization (equal).

## CONFLICT OF INTEREST STATEMENT

The authors declare no potential conflicts of interest.

## Supporting information


**Data S1.** Supporting Information.

## Data Availability

The data that support the findings of this study are available from the corresponding author upon reasonable request.
